# “Floating Island Flap”—A New Technique for the Reconstruction of Full-Thickness Lower Eyelid Defects with Spontaneous Healing (Folded V-Y Island Flap with Orbicularis Oculi Muscle)

**DOI:** 10.3390/jcm13226648

**Published:** 2024-11-06

**Authors:** Andrzej Bieniek, Iwona Chlebicka, Katarzyna Kliniec, Jacek C. Szepietowski

**Affiliations:** 1 University Center for General and Oncological Dermatology, Wroclaw Medical University, 50-367 Wroclaw, Poland; bieniekandrzej2@gmail.com (A.B.); katarzyna.kliniec@gmail.com (K.K.); 2 Faculty of Medicine, Wroclaw University of Science and Technology, 50-370 Wroclaw, Poland; iwonak4wsk@interia.pl

**Keywords:** flap, malignant eyelid tumors, eyelid reconstruction

## Abstract

**Background**: Due to the high incidence of malignant tumors of the lower eyelids and the widespread use of surgery for their treatment, the reconstruction of tissues in this area is a frequent task for surgeons. Methods for restoring full-thickness lower eyelid defects are often invasive, pose a significant risk of complications, and do not provide optimal results. **Methods**: The authors developed a simple technique for the reconstruction of full-wall defects of the lower eyelids. It is a V-Y-type musculocutaneous island flap from the lower eyelid, with the orbicularis oculi muscle folded in the upper part and partially left for spontaneous healing, called by the authors a “floating island flap”. Between 2012 and 2023, 39 patients were operated on using this method. **Results**: Surgical procedures performed under local anesthesia were well tolerated by the patients. The healing process was quick and well accepted. Complications were rare. The most common were: eye irritation and temporary swelling of the flap. In 37 patients, proper protection of the eyeball and good esthetic results were achieved; only in two cases was the cosmetic result assessed as poor. Corneal defects occurred in two cases and healed after conservative treatment. In no cases was reoperation necessary. **Conclusions**: The developed method is simple and effective. It can be widely used in the reconstruction of full-thickness defects of the lower eyelids.

## 1. Introduction

The most common malignant tumor in the Caucasian population—basal-cell carcinoma (BCC)—often develops in the orbital area, in particular in the medial angle and lower eyelid. For this reason, the reconstruction of post-surgical defects of the lower eyelids constitutes a frequent task for surgeons. The authors are also engaged in such surgical treatment; we used to employ various types of reconstructive procedures. These were often extensive, invasive, and their results were not optimal. Shallow and wide defects were also frequently left for spontaneous healing, which usually ensured good results but with noticeable eyelid retraction.

The aim of this work is to present what we have termed the “floating island flap”—a new technique for the reconstruction of full-thickness defects of the lower eyelids—a sliding V-Y musculocutaneous flap from the lower eyelid, with the orbicularis oculi muscle folded in its upper part and partially left for spontaneous healing. We also present the results obtained after such a treatment of 39 cases and discuss them in comparison with other reconstructive methods.

## 2. Materials and Methods

**Figure 1:**
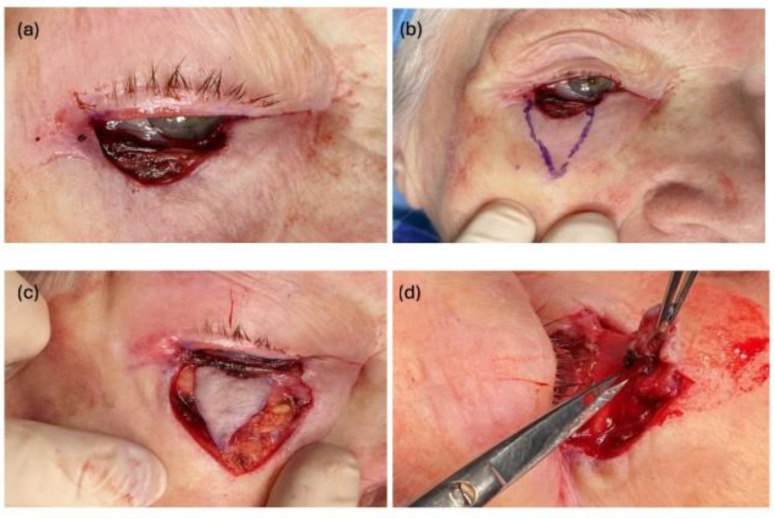

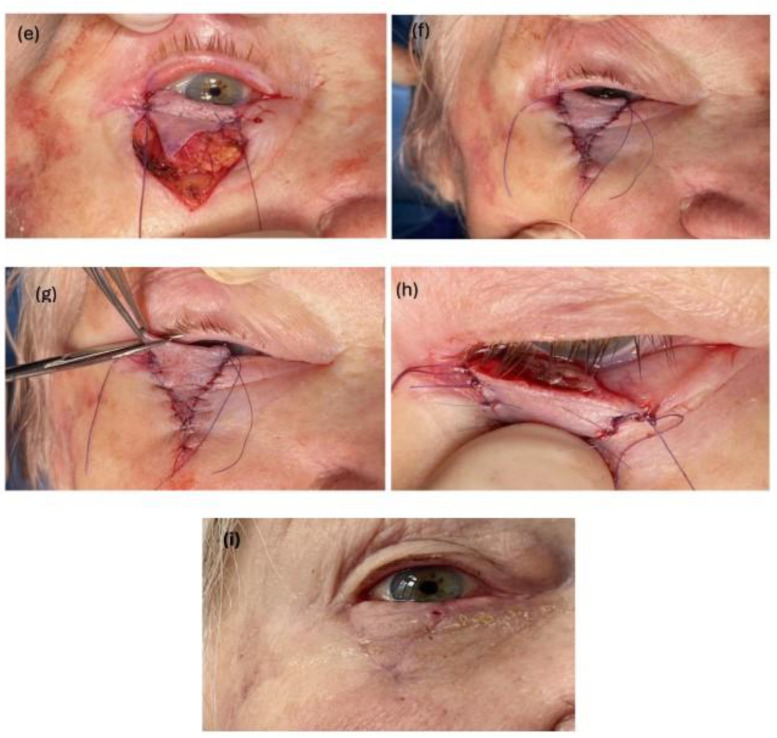
Tissue loss after tumor removal with the use of Mohs micrographic surgery covers its full thickness (outer lamina and inner lamina) (**a**); planning a triangular flap with a width slightly smaller than the defect, reaching the lower edge of the orbit (**b**); the flap after a deep incision covering the skin, subcutaneous tissue, and orbicularis oculi muscle (**c**); the flap is being dissected from above under the orbicularis oculi muscle to approximately 1/3 of its height (**d**); the flap is being sutured to the edge of the eyelid (gray line), leaving its upper portion hanging freely above the edge. We use soft 5-0 braided threads here (**e**); the rest of the flap sutured into the defect with 6-0 monofilament sutures. Long sutures on the edge of the eyelid are to be attached to the skin with tapes (**f**); the free part of the flap during modeling—adjusting to the dimensions of the internal lamina defect (cutting the “corners” of the flap) (**g**); when the eyelid is pulled down, a slit wound becomes visible with the orbicularis oculi at its bottom. This is not subject to modeling and is left intact to increase the stiffness of the eyelid (**h**); a good functional and esthetic result two weeks after the surgery (**i**)—reconstruction of the defect with a “floating island flap” 3 months after the procedure.

All procedures were performed in accordance with the 1964 Helsinki Declaration and its later amendments. The procedure is performed in local anesthesia. Figure 1 shows the case of a 73-year-old woman with BCC of the lower eyelid. Below the lower edge of the defect (Figure 1a), two lines are drawn on the skin, together forming a triangle (Figure 1b), with a width slightly smaller than the defect. Its peak usually reaches the lower orbital rim. A deep incision is made along the lines, through the skin, subcutaneous tissue, and the full thickness of the orbicularis oculi muscle (Figure 1c). The flap is also dissected from above, at approximately 1/3 of its height, under the orbicularis oculi muscle (Figure 1d). Then, the flap becomes very mobile and resembles a “floating island” composed of flaccid tissues, moving freely on the thin orbital septum covering the soft periocular fat. After hemostasis is achieved, the flap is pulled upwards and sutured to the edge of the eyelid (gray line) in such a way that a loose part corresponding to the size of the internal lamina defect remains above it. The authors use single 5-0 braided sutures and leave their ends long, which are later attached to the skin (Figure 1e). Then, the lateral edges of the flap are sutured to the skin of the eyelid, starting from the top, with single 6-0 sutures, consolidating the upward advancement of the tissues (Figure 1f) Because the flap is elastic and based on the flaccid periocular fatty tissue, it can be moved up even a considerable distance (up to 2.5 cm). After completing the suturing, the V-shaped wound turns into a Y, similar to other sliding flaps. Then, the upper, freely hanging part of the flap should be adjusted to the shape of the internal lamina defect by cutting out excess skin (“horns”) of its lateral parts (Figure 1g). Flap modeling does not concern the orbicularis oculi muscle, which should be left intact (Figure 1h). The authors do not suture the upper edge of the flap to the edges of the internal lamina defect; nevertheless, the flap usually remains folded. The flap ensures full-thickness eyelid reconstruction; its upper and folded portion restores the inner lamina of the eyelid (palpebral conjunctiva and tarsus), and its remaining portion—the outer lamina (skin and orbicularis oculi muscle) (Figure 1i).

The described reconstruction was used between 2012 and 2023 in 39 patients ranging in age from 33 to 90 years, including 10 men and 29 women with full-thickness defects of the lower eyelids. Thirty-six cases involved basal-cell carcinoma, one case involved squamous-cell carcinoma, and two cases involved pigmented nevi with full-thickness defects of the lower eyelids. Their width ranged from 12 mm to 35 mm, the depth of the posterior lamina defects ranged from 3 mm to 10 mm.

Follow-up examinations of operated patients were carried out after 1 week, 3 months, and 1 year. The following factors were assessed: the state of postoperative wound healing (delayed healing/wound dehiscence), the presence of flap bruising, swelling, conjunctival hyperemia and eyeball irritation, dry eye syndrome, retraction of the eyelid and its ectropion, corneal erosion (epithelial defects), the level of the patients’ complaints related to the procedure and the postoperative period, and the obtained esthetic results. Dry eye syndrome was diagnosed in the presence of drying out requiring the use of moisturizing drops, as well as a tendency to excessive tearing and hypersensitivity after exposure to light, wind, and cosmetics. A two-level scale (good and bad) was used to assess the acceptance of the procedure, the early postoperative period (by patients), and the esthetic results (by surgeons and patients).

## 3. Results

The results are presented in Table 1.

After one week, 13 patients (33.3%) had conjunctival hyperemia, 5 (12.9%) had flap swelling, 6 (15.3%) had delayed wound healing, and 3 (7.6%) had flap bruising. Patients accepted the course of the surgery and the postoperative period well in 35 cases (89.7%).

**Figure 2:**
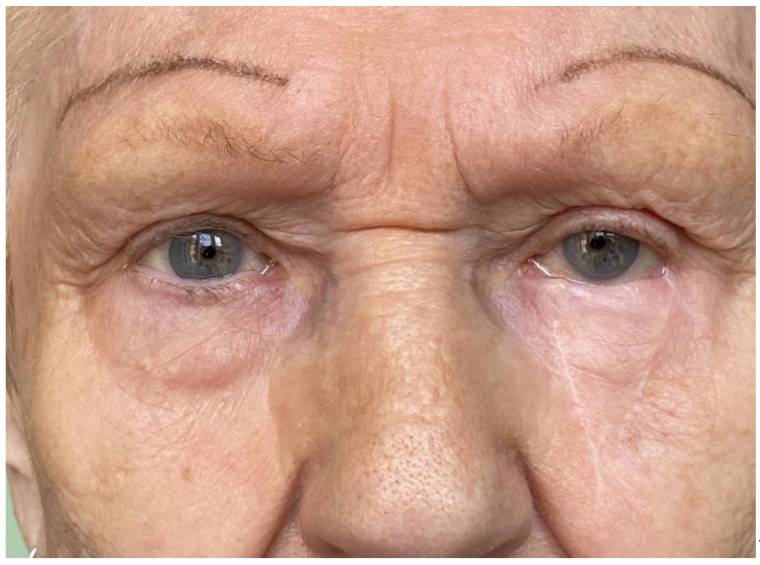
Noticeable lower eyelid retraction two years after basal-cell cancer excision (defect width 22 mm) with V-Y reconstruction in an 83-year-old woman. No further correction was planned at that time.

**Figure 3:**
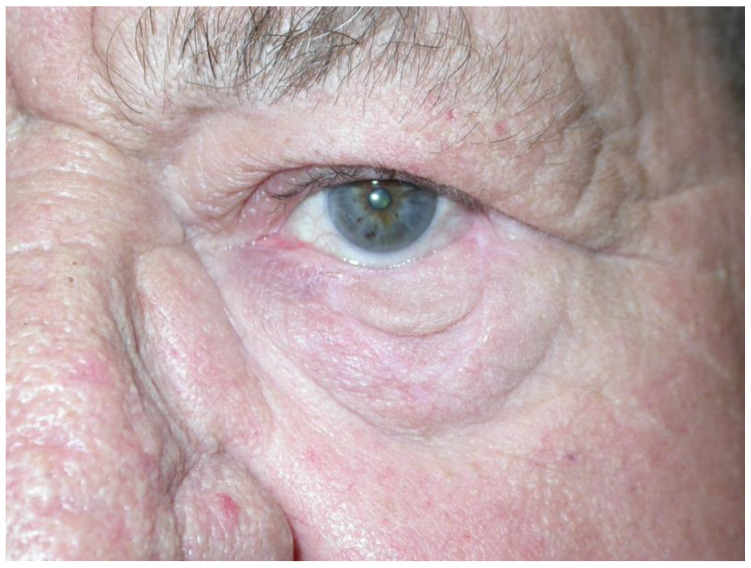
Significant lower eyelid retraction three years after basal-cell cancer excision (defect width 20 mm) with V-Y reconstruction in an 80-year-old man. Further correction was proposed (involving wedge excision of the lateral portion of the lower eyelid); however, the patient accepted the current results.

During subsequent follow-up examinations: after 3 months—4 patients (10.2%) experienced flap swelling (resolving completely within a year); 8 (20.5%) experienced irritation of the eyeball/conjunctival hyperemia, resolved after a year in 4 (10.2%), or slight ectropion of the lower eyelids (also resolving within a year); 12 (30.7%) experienced dry eye syndrome [resolved after a year in 7 (20.0%)]; 32 (82%) experienced various degrees of eyelid retraction [resolved after a year in 27 (77.1%)]; good cosmetic results according to the surgeon were achieved for 32 (82%) patients [after a year for 31 (88.5%)]; good cosmetic results according to the patient’s assessment (after both 3 months and 1 year for 100%). In two cases, defects of the corneal epithelium were found (3 months and 1 year after surgery), which healed within one month as a result of conservative treatment. Significant eyelid retraction occurred in two cases (5.1%)—after 3 months and after a year—when the deepest defects were treated. This condition was considered esthetically unsatisfactory in the surgeon’s opinion. However, because of the patients’ refusals, no corrective procedures were performed. Figure 2 and Figure 3 show cases of patients with eyelid margin retraction.

## 4. Discussion

For many years, we have used various methods to reconstruct full-thickness defects of the lower eyelids. Defects up to about 20% of the eyelid width were usually closed by layered suture. Wider defects, encompassing both laminae of the eyelid, were most often reconstructed separately, using different tissues. The outer lamina was reconstructed using flaps from the cheek and temple, forehead, upper eyelid, or full-thickness skin grafts [1,2,3,4,5]. The inner lamina was restored by conjunctival advancement muco-tarsal flaps from the upper eyelid (according to Hughes [6]), mucosal grafts from the oral cavity and muco-periosteal grafts from the hard palate. To prevent eyelidectropion, we avoided advancement of tissues located directly below the defect.

During procedures involving rotation of the lateral cheek, due to the need to shape the defect in a triangle, we used to remove a significant portion of the healthy tissue in the neighborhood of the defect.

In 2012, for the first time we decided to use this tissue triangle for a two-layer reconstruction of defects of the lower eyelid in the form of an upward, advanced, island flap with the orbicularis oculi muscle, folded in its upper portion. Due to its significant mobility, we called it the “floating island flap”. Initially, we used such flaps for relatively small defects with a minimal loss of the internal lamina; after gaining experience, we also used them for reconstructions of wider and deeper defects. We found that only limited tissue preparation was required, and the procedure was minimally invasive. We also confirmed the good acceptance of the method by patients and its significant effectiveness. For this reason, we gradually began to limit the use of other surgical methods, reserving them mainly for the largest defects.

A few weeks after the procedure, the eyelid shape was usually normal and postoperative scars were hardly visible. Visible differences compared to the healthy part of the eyelid resulted from the lack of characteristic bulging of the eyelash margin (due to the lack of a tarsus), the lack of eyelashes, and slight retraction.

The results of treatment with the new method seem to be better than previously used reconstructive methods; however, we have not undertaken similarly detailed comparative observations. In particular, we noticed the reduction in the complication rate and improved functional and esthetic results.

We believe that it is beneficial to leave the distal (upper) part of the flap without suturing it to the edge of the defect in order to allow secondary intentional healing. This reduces postoperative complaints and prevents eyeball irritation. Moreover, leaving an open, slit-shaped wound in this area results in the conjunctiva creeping onto the “tissue scaffold” formed by the posterior, distal part of the flap devoid of epithelium. As a consequence, this leads to an increase in the area covered by the conjunctiva within the posterior lamina of the eyelid. We feel that this also results in the optimal shaping of postoperative scars, due to their “modeling” on the surface of the eyeball, which constitutes a kind of “template”. Even if the upper part of the flap sometimes remains straight (not folded), due to its increased stiffness, it does not prevent the defect from healing completely within a few weeks. In such cases we even noticed the increased height of the reconstructed eyelids.

**Figure 4:**
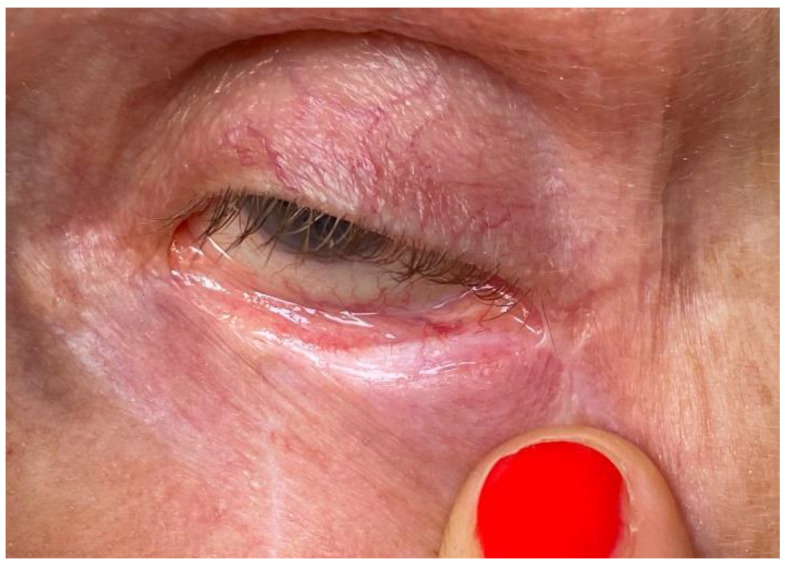
The inner lamina is covered with skin from the folded portion of the flap and epithelium creeping from the neighboring conjunctiva during spontaneous healing.

The inner layer of the eyelid, reconstructed from the folded skin, becomes visible when it is everted. It resembles the conjunctiva—it is shiny, slippery, but paler compared to the surrounding mucosa. Usually, the skin portion of the inner layer of the eyelid is smaller than planned during surgery. This is due to the fact that a significant part of it is covered with conjunctiva, which creeps onto the posterior side of the flap, promoting its epithelialization in the process of secondary intention healing. The scars on the border of the inner part of the flap and the palpebral conjunctiva are thin and flexible. Figure 4 shows the case of an 83-year-old woman after excision of BCC and reconstruction of the defect with a “floating island flap”—3 months after surgery. The value of the partial use of spontaneous healing in surgical reconstructions of the eyelids was also emphasized by other authors [7,8]. They observed spontaneous creeping of the conjunctiva onto cartilage grafts placed within the inner lamina of the eyelid.

Our method “forgives mistakes”. The eyelash margin, which showed unevenness immediately after the removal of sutures, spontaneously leveled out during further healing.

In the vast majority of cases, there is no permanent irritation of the eyeball. In certain cases, the irritation was induced by friction of the turned in eyelashes, growing adjacent to the scar from the lower or even upper eyelid. Eyelash electrolysis resulted in the resolving of irritation. However, ocular irritation by eyelashes may develop even after pure spontaneous healing, which seems to be the least invasive “reconstructive method” [9]. In a few cases, we also found eye irritation caused by the velus hairs of the eyelid skin, which was successfully treated by laser epilation. Nevertheless, this did not seem to pose a serious problem, like after palpebral reconstructions using thick and hairy skin transposed from the cheeks [10].

Our technique often does not provide complete recreation of the eyelid height, and slight retraction occurs in most cases. However, this does not have a significant negative impact on the esthetic result. For this reason, we believe, like other authors [9,11], that a certain degree of retraction should not be treated as a failure of reconstruction. It should be borne in mind that the so-called scleral show, i.e., the visibility of the sclera below the edge of the iris, occurs very often in the healthy population [12].

Some doubts may arise as to whether the blood supply to this complex flap, dependent on small perforating vessels within the relatively narrow pedicle, is always sufficient. We observed that the risk of circulatory disorders is greater in narrow flaps, where bruising was more common. They disappeared after 5–10 days, and in no case did flap necrosis occur. However, lymphatic stasis is common. Swelling usually starts about 1–2 weeks after the procedure and may last for many weeks. Massages performed by patients make them resolve faster. In no case did these swellings prove to be permanent.

Some concerns may also be raised about the functioning of the lower eyelid after dissecting and moving a significant part of the orbicularis oculi muscle. However, after a few months, the eyelid mobility returned almost to its initial state.

Initially, we designed a flap with a width corresponding to the full width of the defect, similarly to Seth et al. [13]. However, we noticed that better results were achieved when the flap was narrower than the defect, which resulted in increased horizontal tension and a higher position of the eyelid margin [14]. An analogous technique of lifting the lash margin by increasing the tension is typically used in eyelid retraction operations using the Kuhnt–Szymanowski method or in postoperative reconstructions [14,15,16]. For this reason, we currently perform V-Y flaps with a width corresponding to approximately 80% of the defect.

We most often make flaps the peak of which is located near the lower edge of the orbit; only in the most extensive defects was it located lower—even at the level of the nasal wing, similarly to the case described by Seth and others [13]. However, we do believe that this is most often unnecessary. Similar triangular flaps of a small size have also been used by other authors with good results [14]. However, these were unfolded flaps, used to reconstruct only the outer lamina.

The best results can be expected when the defect of the inner lamina does not cover the full height of the eyelid. According to our observations, this corresponds to the shape of lower eyelid defects after the removal of tumors with use of Mohs micrographic surgery [13].

It is necessary that the upper part of the flap is prepared under the orbicularis oculi muscle, and not under the skin. This makes the flap thicker, ensures more effective filling of the defect, and increases the stiffness of the eyelid.

In the commonly used Mustardé technique, the defect is shaped into a triangle, discarding healthy tissues [1]. Our method may be described as lossless, because those spared tissues are employed for reconstruction. This means that, if reoperation is needed, the Mustardé method can still be used.

When discussing alternative reconstruction methods, it is impossible to ignore the spontaneous healing of defects. The method is minimally invasive, allows for the closure of even extensive tissue defects, and its esthetic and functional results are good [9,11]. However, its significant drawback is the significant lowering of the eyelid margin. The “floating island flap” technique can be considered a specific improvement of spontaneous healing, in which the palpebral conjunctiva creeps onto the tissue scaffold pulled up from below, which ensures better reconstruction of the eyelid height.

Our technique is inconsistent with the commonly recommended practice of posterior eyelid lamina reconstruction with mucosa [17]. In our method the eye globe is in contact with the skin, not the mucous membrane. It should be noted, however, that this rule is also broken in other reconstructive methods. For example, Uemura et al. [8] used skin-cartilaginous grafts from the auricle, which were in contact with the eyeball.

The abandoning of the separate reconstruction of the posterior eyelid lamina is beneficial, not only because of the simplicity, but also safety. The above-mentioned procedures pose a considerable risk of complications, and their results are often suboptimal [18,19,20,21]. It is also beneficial that our method is a one-stage one, unlike e.g., Hughes’s or Tripier’s methods, which require secondary excisions of the flap pedicles [21].

We do not use additional stiffening of the eyelid with cartilage, because the skin, fat, and muscle contained in the flap reproduce its structure well. A similar method of eyelid reconstruction was also used by Garces et al. [14]. The eyelid reconstruction technique they described using two V-Y flaps—a musculocutaneous and a conjunctival flap—made it possible to obtain good functional and esthetic results.

What may also raise objections is the use of tissues transferred directly from below, as well as the transsection of the lateral edges of the flap with the horizontal skin tension lines of the orbital area. These conditions increase the risk of downward displacement of the eyelid, its eversion, and ectropion. Due to fear of such complications, special modifications of the V-Y flaps have been developed to move them laterally rather than directly inferiorly [16,22,23,24]. However, in our material, such complications did not occur.

Triangular flaps, consisting of skin and deeper tissues, advanced longitudinally for reconstruction of the lower eyelid, have been previously described by many other authors [13,14,16,25,26,27,28,29,30,31]. However, they were only used to reconstruct the outer lamina of the eyelid. During a literature review, we found that a surgical technique similar to ours called VYFF (V to Y Fold over Flap) had recently been presented by Seth et al. [13]. Apart from this single case, however, full-thickness reconstruction of the eyelid (both laminae) by folding the distal part of this flap has not been described.

We acknowledge the limitations of this study. First, as an observational study focused on evaluating a method developed to improve surgical outcomes, there was sometimes a lack of detailed data and no control group. Secondly, due to methodological differences in studies describing other techniques, direct comparisons were not feasible. Factors such as variations in interpreting irritation, patient satisfaction, eyelid positioning, and differences in surgical approaches among surgeons made comparisons challenging. This study included only an intuitive comparison to older methods previously used by our team, and the new method appeared to show improved results.

## 5. Conclusions

Based on 39 operated-on patients, we believe that the reconstruction of lower eyelid defects using a “floating island flap”—a folded VY cutaneous-muscular flap from the lower eyelid with the orbicularis oculi muscle partially left for spontaneous healing within the inner lamina—is a simple and minimally invasive technique that ensures high patient acceptance, a low risk of complications, and favorable functional and esthetic results.

## Figures and Tables

**Table 1:** The results of the reconstruction of full-wall defects of the lower eyelids using a “floating island flap”—a folded V-Y musculocutaneous flap with the orbicularis oculi muscle, partially leaving the defect for spontaneous healing.

Results	1 Week		3 Months		1 Year	
	N/39	%	N/39	%	N/39	%
Delayed healing/wound dehiscence	6/39	15.3	X	X	X	X
Flap bruising	3/39	7.6	0/39	0	0/39	0
Flap swelling	5/39	12.8	4/39	10.2	0/39	0
Irritation of the eyeball/conjunctival hyperemia	13/39	33.3	8/39	20.5	4/39	10.2
Dry eye syndrome	X	X	12/39	30.7	7/39	17.9
Eyelid margin retraction	X	X	32/39	82.0	27/39	69.2
Eyelid ectropion	X	X	6/39	15.3	0/39	0,0
Corneal epithelial defects	X	X	1/39	2.5	1/39	2.5
Good acceptance of the procedure and the healing period by patients	35/39	89.7	X	X	X	X
Good cosmetic result according to the patients	X	X	39/39	100	39/39	100
Good cosmetic result according to the surgeon	X	X	37/39	94.8	37/39	94.8

X—the given parameter was not tested at that time.

## Data Availability

Detailed data is available from the corresponding author on request.
